# Effect of Selenium Sources on Laying Performance, Egg Quality Characteristics, Intestinal Morphology, Microbial Population and Digesta Volatile Fatty Acids in Laying Hens

**DOI:** 10.3390/ani11061681

**Published:** 2021-06-04

**Authors:** Aliyu Ibrahim Muhammad, Dalia Alla Mohamed, Loh Teck Chwen, Henny Akit, Anjas Asmara Samsudin

**Affiliations:** 1 Department of Animal Science, Faculty of Agriculture, Universiti Putra Malaysia, Serdang 43400, Malaysia; aliyudambam@gmail.com (A.I.M.); tcloh@upm.edu.my (L.T.C.); henny@upm.edu.my (H.A.); 2 Department of Animal Science, Faculty of Agriculture, Federal University Dutse, Dutse 7156, Nigeria; 3 Department of Animal Nutrition, Faculty of Animal Production, University of Khartoum, Khartoum 321, Sudan; dalida983@yahoo.com

**Keywords:** bacterial selenoproteins, laying performance, egg quality characteristics, intestine histomorphology, caecum microbes, digesta volatile fatty acids, laying hens

## Abstract

**Simple Summary:**

Deficiency in microminerals (e.g., selenium) is a global issue, with a consequent manifestation of health-related challenges in both humans and animals, and is associated with poor animal production and reproductive performance. Due to the health benefit of selenium, dietary supplementation of organic selenium for caecum microbes can be important for balance, maintenance, and performance requirements. Food products containing selenium (eggs) are often referred to as “functional foods”, and are simple to produce from organic sources (selenium). Dietary supplementation with bacterial selenoproteins improves performance index, egg quality parameters, intestinal villus height, and increases the population of beneficial, and decreases harmful caecum microbes in hens, according to the current study. Therefore, it can be used to effectively produce Se-rich eggs as a functional food, while also supporting the caecum microbiome.

**Abstract:**

The use of toxic and less bioavailable inorganic selenium can now be supplemented with an alternative organic source from bacterial species in nutrition for human and animal benefit. This study investigated the effects of selenium sources on laying performance, egg quality characteristics, intestinal morphology, caecum microbial population, and digesta volatile fatty acids in laying hens. One hundred and forty-four Lohman Brown Classic laying hens, at 23 weeks of age, were divided into four experimental groups (36 hens in each), differing in form of Se supplementation: no Se supplementation (Con), 0.3 mg/kg of inorganic Se in the form of sodium selenite (Na_2_SeO_3_), 0.3 mg/kg of organic Se from selenium yeast (Se-Yeast), and 0.3 mg/kg of organic Se from *Stenotrophomonas maltophilia* (bacterial organic Se, ADS18). The results showed that different dietary Se sources significantly affected laying rate, average egg weight, daily egg mass, feed conversion ratio (FCR), and live bodyweight (LBW) (*p* < 0.05). However, average daily feed intake and shell-less and broken eggs were unaffected (*p* > 0.05) among the treatment groups. The findings revealed that selenium sources had no (*p* > 0.05) effect on egg quality (external and internal) parameters. However, eggshell breaking strength and Haugh unit were significantly (*p* < 0.05) improved with organic (ADS18 or Se-yeast) Se-fed hens compared to the control group. In addition, egg yolk and breast tissue Se concentrations were higher (*p* < 0.05) in the dietary Se supplemented group compared to the control. Intestinal histomorphology revealed that hens fed ADS18 or Se-Yeast groups had significantly (*p* < 0.05) higher villi height in the duodenum and jejunum compared to those fed Na_2_SeO_3_ or a basal diet. However, when compared to organic Se fed (ADS18 or Se-Yeast) hens, the ileum villus height was higher (*p* < 0.05) in the basal diet group; with the lowest in the SS among the treatment groups. A significant increase (*p* < 0.05) of *Lactobacilli* spp. and *Bifidobacteria* spp., and a decrease of *Escherichia coli* and *Salmonella* spp. population were observed in the organic (ADS18 or Se-yeast) compared to inorganic supplemented and control hens. The individual digesta volatile fatty acid (VFA) was significantly different, but with no total VFA differences. Thus, bacterial selenoprotein or Se-yeast improved the performance index, egg quality characteristics, egg yolk and tissue Se contents, and intestinal villus height in laying hens. Moreover, caecum beneficial microbes increased with a decrease in the harmful microbe population and affected individual cecal volatile fatty acids without affecting the total VFA of the laying hens digesta.

## 1. Introduction

The need for healthy and quality animal products (meat, egg, and milk) fortified with micronutrients that support the health of the growing human population globally is paramount and increasing [1]. Poultry products are also the second most widely consumed globally, accounting for around 30 percent of meat production. Selenium (Se) has been recognized as an essential micro-element for improving the performance, health, and antioxidant system of poultry [2]. It is an integral part of more than 25 selenoproteins, such as the glutathione peroxidase enzyme, that participate in the regulation of cellular functions in the body. It is an important constituent of glutathione peroxidase (GSH-Px), an antioxidant enzyme that acts primarily to prevent free radical (ROS) formation, and is involved in the metabolic regulation of various hormones, such as thyroid hormones [3,4]. Improving animal antioxidant capacity, immunoregulation, and production and fertility performance, thyroid hormone metabolism, and subduing the harmful effects of free radicals (detoxification) were notable among the benefits of Se [5,6,7,8].

Dietary Se deficiency, on the other hand, causes a wide range of diseases, including Keshan disease, Kaschin–Beck disease, and goiters in humans [9,10,11,12]; and weak immunity and reduced egg production, liver necrosis, poor fertility, white muscle disease, muscular dystrophy, and exudative diathesis in livestock animals [4,8,13,14]. Excess Se intake, however, is toxic [13], and the low bioavailability of the inorganic form of Se discourages its dietary use in nutrition because of its toxicity [14]. Organic and inorganic are the main sources of Se used in diets. They both impart a positive effect on poultry performance; however, many findings have revealed higher absorption and utilization with organic [15,16,17,18] than inorganic Se. Kieliszek and Blazejak [16] reported Se assimilation differs in its form, with about 90–95% and 80–85% organic and inorganic, respectively.

Consequently, selenium supplements, sometimes as chemoprevention, are currently becoming mainstream for nutritional requirements. The organic forms of Se are most recommended for satisfying the dietary requirements of improved performance compared to inorganic forms [19,20]. Furthermore, there has been lower environmental pollution as a result of Se excretion [21]. Therefore, the quest for nutrition or supplements of higher organic Se quality through low-cost and uncomplicated techniques has acquired growing consideration year-on-year. This has become imperative for expanding Se content via enriched-foods to humans for its health benefits. For instance, organic Se was found to be deposited significantly and to improve egg quality [22]. Antioxidants such as vitamin E, carotenoids, flavonoids, and selenium are abundant in our food [23]. Modified or enriched eggs are those in which the content has been modified from standard eggs, and are classified as nutritionally enhanced eggs, value added eggs, or processed eggs [24]. Selenium–enriched eggs with a specific selenium content may be used as a functional food to increase human selenium intake and, thereby, the amount of selenium in the body [25,26,27]. Chicken eggs are a common food that is eaten by people of all ages in almost every country [28]. Selenium-enriched eggs contain above-average levels of Se in the form of selenomethionine (Se-Met); an essential amino acid needed for optimal physiological function in the body [28]. The selenium levels in a regular egg and a selenium-enriched egg are around 11 µg and 32.6 µg, respectively; hence, eating two selenium-enriched eggs per day will provide over 70% of the daily dose of selenium recommended for humans [29].

The small intestine is an organ situated in the gastrointestinal tract and which is mainly involved in nutrient digestion and absorption, thus improving intestine function results in an increase in the utilization of nutrients, which in turn improves the general performance of animals [30]. The development of the chicken gastrointestinal tract is linked to the characteristics of the small intestine as a site of nutrient absorption and reflects the efficiency of the used nutrients in poultry [31,32]. The general well-being of poultry is also centered on the structure of the intestine and intestinal microbial population [33,34]. The small intestine comprises three structural parts; the duodenum, jejunum, and ileum. Wang and Peng [35] reported crypts and villi of the small intestine as part of the absorptive epithelium that plays a significant role in the final phase of nutrient digestion and assimilation.

Intestinal development can be evaluated by measuring the crypt, the region where new intestinal cells are developed, as well as the height and surface area of the villus, to determine the area available for digestion and absorption [32,36]. However, there is a lack of reports linking the effects of dietary microelements and especially that of selenium on the development of the gastrointestinal tract of poultry. The morphological changes in intestinal villi in broilers were found to rely on the presence of digested nutrients in the small intestinal lumen [37]. Awad et al. [38] observed an increase in broiler villus height to crypt depth ratio and villus height in duodenum and ileum, while the duodenal crypt depth remained unaffected with dietary supplementations of synbiotics and probiotics. Chichlowski et al. [38] reported that probiotics containing *Lactobacillus* of *Bifidobacterium thermophilum* and *Enterococcus faecium* increased the jejunal villus height and decreased the villus crypt depth compared with other treatments. Moreover, shorter and thinner villi were associated with toxin adverse effects [39], but longer villi represented an improvement in nutrient efficiency, as reported by male layers after dietary addition of *Bacillus subtilis* var. *natto* [40] and in broilers after adding *E. faecium* or *Eubacterium sp.* [39,41].

The intestinal microbiome is a complex system of distinct biochemical pathways and is thus referred to as a different organ that is superior to the liver in conversion function [42]. In a mutualistic relationship with the intestine host, the microbial population has coevolved and has the capacity to interact with other species and with the host [43]. The microbiome is closely involved in host health, including regulating cell proliferation, gut immunity, and the synthesis of essential nutrients needed for both the microbes and the host [44,45]. Therefore, recognizing to what degree the gut population of each organism spp influences nutrient bioavailability is a major challenge in confirming the effectiveness of new dietary supplements [42]. Dietary supplementations influencing the gut microbiota have been proposed to have considerable potential as alternative methods for impacting human health [46]. The use of prebiotics could be one of the key strategies for regulating and modulating the gut microbiota through dietary microelements [47]. The microorganisms in the gut are microelement-sensitive. For some species of microbes, selenium is essential for various metabolic functions, although, on the other hand, it may be toxic at low concentrations [48,49]. The composition of the intestinal microbiota can therefore be modulated by changes in the dietary microelements. Increases in the population of the beneficial community may enhance the host bird’s productivity [50]. Therefore, a realistic approach to host health safety is to increase the number of beneficial bacteria to prevent the colonization of harmful bacteria [51,52]. Dietary selenium supplementation, for example, influences the intestinal composition of microflora and gastrointestinal tract colonization, which, on the other hand, affects the host selenium status and selenoproteome expression [47].

The supplementation of Se using different species of microorganisms, such as bacteria, is a recent development for providing the nutritional benefits of microelements [53]. There have been diverse strains of bacteria used in the microbial reduction processes that can assimilate and transform the inorganic form and be retained in cells as organic Se and in the form of selenoprotein [54]. In line with the aforementioned reasons, *Stenotrophomonas maltophilia* bacteria (ADS18) was chosen as a selenoprotein with higher Se concentrations in its cells, based on their ability to accumulate absorbed Se [55,56]. While Se can improve hen performance, there is little scientific evidence on the effect of this new organic Se source on layers (egg-laying hens). No published research on the effect of bacterial organic Se (ADS18) on laying performance, egg quality characteristics, intestinal morphology, caecum microbial population, and digesta volatile fatty acids in laying hens has been reported. As a result, the present study was to compare the efficacy of bacterial organic Se (ADS18) as an alternative organic Se source to inorganic Se on laying performance, egg quality characteristics, intestinal morphology, caecum microbial population, and digesta volatile fatty acids.

## 2. Materials and Methods

### 2.1. Preparation of Organic Selenium from Bacteria (Stenotrophomonas maltophilia)

*Stenotrophomonas maltophilia* (ADS18) was isolated from hot spring water for its higher seleno-amino acids contents (mainly Se-meth). The stock culture was prepared at the Laboratory of Microbiology, Department of Animal Science at the Faculty of Agriculture, Universiti Putra Malaysia (UPM), following the method described by Dalia et al. [55,56].

### 2.2. Animal Ethics

This study received the approval of the Institutional Animal Care and Use Committee of the Universiti Putra Malaysia (UPM/IACUC/AUP-R063/2018). All procedures were carried out under the guidelines and regulations stipulated for the administration affairs relating to experimental animals.

### 2.3. Experimental Animals, Design, and Treatments

A total of one hundred and forty-four 18-week-old Lohman Brown hens (initial live weight 1714 ± 185 g) were purchased from a commercial farm in Kuala Selangor, Selangor, Malaysia. The pullets were reared in an open ventilated layer-house and A-shape two-tier stainless-steel cages, with a bird per cage, at the Poultry Unit, Universiti Putra Malaysia, Serdang. The size of the cages was 30 cm by 50 cm by 40 cm (width depth height). A corn and soya bean-meal basal pre-lay and layer diet was prepared using FeedLIVE software based on the Lohman management guide (2018) Table 1 and Table S1. The pre-lay diet was provided to the pullets from 18 to 22 weeks, while the layers diet continued afterward. At 23 weeks of age, the hens were randomly divided into four homogenous groups (36 hens in each), with six replicates (6 hens per replicates) differing in the form of Se added to the standard diet. The hens in group I were fed diets without Se supplementation, the hens in group II were supplied with 0.3 mg/kg of inorganic Se in the form of sodium selenite, the hens in group III were supplied with 0.3 mg/kg of organic Se from Se-yeast SelPlex (Altech Inc., Lexington, KY, USA) (SY), and the hens in group IV were supplied with 0.3 mg/kg of organic Se from *Stenotrophomonas maltophilia* (bacterial organic Se, ADS18). Selenium concentration in the diet without Se supplementation, with inorganic, with organic Se-yeast, and bacterial organic Se (ADS18) was determined to be 0.031, 0.312. 0.320, and 0.339 mg/kg, respectively. The organic selenium content of *Stenotrophomonas maltophilia* (ADS18) product was 4.51 µg/g. The Se content of the diets was analyzed using Inductively Coupled Plasma Mass Spectrometry (ICP.MS) Table 2. The total Se content, organic Se content, quantification methods of *Stenotrophomonas maltophilia* (ADS18) product was described in previous studies [55]. The hens were limited to 120 g/hen/day to reduce the feed-selection behavior commonly spotted in laying hens. Feed was given once a day (07:00–08:00) and access to water was allowed ad libitum. In the experimental phase, the ambient temperature was approximately 30 ± 5 °C. The lightening schedule was practiced with 16-h light and 8-h dark, with light beginning at 17:00 local time and according to the Lohman management guide (2018). The experiment lasted for sixteen weeks (112 days), except for four weeks of adaptation.

### 2.4. Slaughtering and Sampling

Twelve hens were selected randomly from each dietary treatment (2 hens from each replicate), slaughtered at 39 weeks of age according to the Halal procedure described in the Malaysian Standard [57], and breast meat samples for Se determination were collected and stored at −80 °C for further analysis. A total of 48 samples were used, with twelve samples per treatment group. At the end of the 16-week experiment, four representative eggs per replication were randomly sampled and collected for the egg yolk Se assay.

### 2.5. Measurements

#### 2.5.1. Growth and Laying Performance

Individual hen body weight (BW) and feed intake (FI) were recorded weekly, where changes in body weight and feed conversion ratio (FCR) were calculated. Laying FCR was computed as g feed consumption divided by g egg mass (g feed/g egg mass). For laying performance, daily egg number and egg weight were recorded for each hen, and egg production (laying rate) was assessed weekly as egg number/hen/period for each treatment. Egg mass was calculated by multiplying egg numbers by average egg weight. The number of normal eggs, soft-shelled or cracked eggs, egg weight, and mortality and morbidity of hens per cage were documented and recorded daily.

#### 2.5.2. Eggs Quality Traits

Every two weeks during the experimental periods, six representative eggs per replicate were sampled to determine egg quality (external and internal) characteristics.

##### Eggs Physical Characteristics

Eggs laid at different sampling points were collected, weighed using a digital balance, and the physical characteristics of eggs (egg length and width (mm)) were measured with a digital vernier caliper (150 Mm). After washing and drying overnight at 60 °C, the eggshell (g) was weighed. The indexes of the egg shape and surface area were calculated as described [58,59]. Similarly, shell thickness was measured at three different locations (middle, and broad and narrow ends) using a micrometer gauge (Digimatic 0–25 mm 0.001 mm, Mitutoyo Inc., Kawasaki, Japan) with spherical tips. Eggshell breaking strength (N) was measured by quasi-static compression using a testing machine (model 5542, Instron, Norwood, MA, USA) fitted with a 500-N load cell and equipped with a food texture fixture compression anvil (Catalog No. 2830-009, Instron, Norwood, MA, USA) [58]. Breaking strength was measured as the maximum force required to fracture each egg at a compression speed of 5 mm/min [58].

##### Eggs Internal Characteristics

Measurements of the internal egg parameters were obtained after taking the external observations by breaking the eggs and depositing the content (albumen and yolk) on the black tray for the Haugh unit and color measurement. The egg content was separated using a plastic egg separator tool, which allowed content separation. The yolk color and Haugh unit were measured using an egg analyzer from Orka Food Technology Co., Ltd. (Ramat HaSharon, Israel). Weight and height of albumen and yolk, and yolk to albumen ratio were obtained as described [60].

### 2.6. Selenium Content Determination

To determine the Se content in egg yolks, the egg yolk samples were freeze-dried (Labconco Free Zone plus 6, Kansas, MO, USA) at −50 °C for 72 h and ground to a powder [61,62]. Briefly, approximately 0.5 g egg yolk or tissue was digested in 5 mL of concentrated HNO_3_ (Sigma-Aldrich, St. Louis, MO, USA) and 3 mL H_2_O_2_ (EMSURE^®^ ISO, Merck, Darmstadt, Germany). Then, the digest was diluted with deionized water to a final volume of 10 mL. The Se concentration was determined using a Perkin Elmer DRC-e ICP-MS with calibrations performed every 20 samples.

### 2.7. Histomorphology of the Small Intestine 

The intestinal parts (duodenum, jejunum, and ileum) were identified and isolated as described [63]. Briefly, they were defined anatomically as follows; duodenum length was from the gizzard (duodenum ostium) to the beginning of the mesentery (duodenum loop), jejunum length begins from the distal point of insertion of the mesentery to an average of 5 cm before Meckel’s diverticulum), and lastly the ileum length marks 5 cm above Meckel’s diverticulum to the ileocecal junction. They were excised carefully and phosphate-buffered saline was used to flush and rinse the tissue samples before storage in formalin (10%). All the samples were trimmed to the recommended size (4–5 µm thick) before dehydration in an automated tissue processor (Leica ASP 3000, Wetzlar, Germany) for 16 h. Thereafter, the tissue samples were all embedded using a paraffin-embedding system (Leica ASP 3000, Wetzlar, Germany). A rotary microtome machine (Leica ASP 3000, Wetzlar, Germany) was used to excise each tissue sample at the standard recommended size of 4 µm, they were put in warm or hot water to be flattened, carefully fixed on glass slides, and heated at 57 °C until dry. The tissue sections were stained using hematoxylin and eosin protocols, and thereby the slides were viewed under a light microscope (Leica DM LB2, Wetzlar, Germany) with a fitted digital camera (Leica DFC 295, Wetzlar, Germany). For every six replicates, six villi sections were measured per slide per intestinal sample, thus amounting to a total of 36 measurements per sample. Villus height was measured from the tip of the villi to the villus crypt junction and crypt depth was defined as the depth of the invagination between two villi; both villi height and crypt depth were determined using Image-Pro Plus software connected to the light microscope as described by Touchette et al. [64]. The histomorphometry parameters analyzed were villus height and crypt depth, with villus height to crypt depth ratio (H:D) [65]. The presence of an intact lamina propria and villi, which were perpendicularly sectioned via the midline axis, was the criterion for histological section examination.

### 2.8. Analysis of Caecum Microbiome (Bacteria) Using Real-Time PCR

At the slaughter point, the hen digesta in the ceacum was squeezed and collected into 2 mL Eppendorf tubes, kept on ice, and immediately transferred and stored for microbial population quantification at −20 °C. The procedure reported in [66,67,68] was followed for establishing the population of total bacteria, *Lactobacillus* spp., *Bifidobacteria* spp., *Enterococcus* spp., *Enterobacteria* spp., *Escherichia coli*, and *Salmonella* spp. The genomic DNA materials were extracted from the ceacal digesta content using QIAamp^®^ DNA Stool Mini Kits (Qiagen, Hilden, Germany), following the manufacturer’s instruction. DNA integrity and quality were checked using Thermo Multiskan^®^ GO (Thermo Scientific, Waltham, MA, USA). Serial dilution of PCR products from the pure cultures of the target bacteria was used for calibration curve construction after amplifying a known amount of the target bacteria. The qPCR reaction was carried out using a master mix, QuantiNova SYBR Green PCR Master Mix (Cat. No. 208054). Sequences of primers and the annealing temperature of each targeted ceacal bacteria used in this study are shown in Table 3. Each reaction volume consisted of 10 µL SYBR Green Master Mix, 1 µL of 10 µM forward primer, 1 µL of 10 µM reverse primer, 1 µL of DNA samples, and 7 µL of nuclease-free water. The qPCR assay was run with a Bio-Rad CFX Manager™ 3.1 real-time PCR system (Bio-Rad, Hercules, CA, USA), and by utilizing optical grade plates as follows: the cycling conditions consisted of the first step with PCR initial heat activation (denaturation) at 95 °C for 2 min, then 2-step cycling by 40 cycles of denaturation at 95 °C for 5 s, combined primer annealing/extension at 55 °C for total bacteria, 58 °C for *Lactobacilli* spp., 60 °C for *Bifidobacteria* spp., and 50 °C for *Salmonella* spp., *E. coli*, *Enterococcus* spp., and *Enterobacteria* spp. for 10 s, respectively. The melting curve analysis step was set and performed at the last cycle of each amplification to verify its specificity.

### 2.9. Volatile Fatty Acid Determination from Hen’s Cecal Digesta 

The concentration of volatile fatty acid (VFA) in the hen feces was determined by a modified method explained by Kareem et al. [72]. Approximately one gram of feces was weighed and stored at −20 °C. A 24% metaphosphoric acid was freshly prepared and diluted in 1.5 M 98% H_2_SO_4_ (Emsure^®^ Merck, Darmstadt, Germany), and 1 mL was added to each sample. The mixture was left overnight at room temperature, and centrifuged at 10,000× *g* for 20 min at 4 °C after. The supernatant was harvested, filtered, and transferred into a 1.5 mL screw-capped clear glass vial (Supelco, Inc, Sigma-Aldrich, St. Louis, MO, USA) and stored at −20 °C. An internal standard (Sigma-Aldrich Chemical Co., St. Louis, MO, USA) of 20 mM 4-methyl-valeric acid was used. A 0.5 mL clear supernatant was mixed with an equal volume of 4-methyl-valeric acid steadily (Sigma-Aldrich Chemical Co., St. Louis, MO, USA) as the internal standard. The ceacal VFA were analyzed using a 6890 N Network GC System gas chromatograph (Agilent Technologies) as described by Kim et al. [73]. The VFA separation was performed on a Quadrex 007 Series (Quadrex Corp., New Haven, CT, USA) bonded phase fused silica capillary column (15 m, 0.25 mm ID, 0.25 µm film thickness), used in a chromatograph, with a 6890 N Network GC System gas chromatograph (Hewlett-Packard, Avondale, PA, USA) equipped with a flame ionization detector. Purified nitrogen was the carrier gas at a flow rate of 60 mL/min, with the column temperature set at 200 °C and injector and detector temperature set at 230 °C. An external commercial standard (Sigma-Aldrich Chemical Co., St. Louis, MO, USA) of 20 mM acetic and 10 mM each of propionic, butyric, isobutyric, valeric, isovaleric, and 4-methyl-valeric acids were used for the identification of sample peaks. The molar concentration of VFA was identified per single point of the standards (internal and external).

### 2.10. Statistical Analysis

The data were subjected to a completely randomized design and the data obtained were analyzed using one-way analysis of variance (ANOVA) by a general linear model (GLM) procedure in SAS software 9.4 Version (SAS Institute Inc., Cary, NC, USA). The statistical model used was: Y_ij_ = *µ* + T_i_ + e_ij_. Where Y_ij_ is the mean of the j-th observation of the i-th treatment; *µ* is the sample mean; T_i_ is the effect of the i-th treatment; and e_ij_ is the effect of the error. Histogram distribution and quantile-quantile (Q-Q) plots of the model were used for the assumption of normality. Duncan multiple range test was used to separate means at *p* < 0.05 significance level. The results are presented as mean ± SEM in all tables.

## 3. Results

### 3.1. Laying Performance

Dietary supplementation of different selenium sources on laying rate, average egg weight, daily egg mass, average daily feed intake, FCR, live body weight gain, and shell-less and broken egg rate are summarized in Table 4.The Se supplemented groups had a higher (*p* < 0.05) hen laying rate when compared to the control group, with the highest value recorded in the ADS18 group. However, average egg weight and daily egg mass were similar for all treatment groups, with the exception of ADS18-fed hens, which had a higher average egg weight and daily egg mass when compared to the control group. Dietary Se supplementation had no effect (*p* > 0.05) on average daily feed intake, shell-less, and broken egg rate. Feed conversion ratio was improved (*p* < 0.05) in the bacterial organic (ADS18) Se supplemented group. In comparison to inorganic sodium selenite and the basal diet group, dietary organic (ADS18 or Se-yeast) Se improved hen live body weight (LBW).

### 3.2. Egg Quality Characteristics

The effects of different Se sources on the external and internal characteristics of chicken eggs are described in Table 5 and Table 6, respectively. The findings show that selenium sources had no (*p* > 0.05) effect on egg quality (external and internal) parameters. The eggshell breaking strength and Haugh unit, on the other hand, were significantly (*p* < 0.05) affected. Organic (ADS18 or Se-yeast) Se-fed hens had higher eggshell strength compared to the control groups. Similarly, dietary organic Se supplementation (ADS18 or Se-yeast) substantially increased the Haugh unit as compared to the control group.

### 3.3. Selenium Concentration in Egg Yolk and Tissue

The Se concentrations of egg yolks and tissue are summarized in Table 7. The Se dietary treatments resulted in significantly different Se concentrations in egg yolk. A higher concentration of Se in the yolk was observed in organic Se (ADS18 and SY) supplemented diets, which were significantly (*p* < 0.05) different from the SS and Con groups. Similarly, the meat Se concentration was greatest in hens supplemented with ADS18 and Se-yeast, followed by SS and then Con.

### 3.4. Small Intestine Histomorphology

The villus height, crypt depth, and villus height (VH):crypt depth (CD) ratio of the duodenum, jejunum, and ileum of hens fed different Se treatments are shown in Table 8. Compared to the Con group, supplementation with Se (except ileum) increased (*p* < 0.05) the villi height in the duodenum and jejunum, with organic Se contributing greater (*p* < 0.05) effects than sodium selenite. However, the ileum villi height in the Con group was higher than in the Se treatment groups. Villi height values in the bacterial selenoproteins (ADS18) and Se-yeast groups were comparable. Hens supplemented with Se diets resulted in lower (*p* < 0.05) jejunal and ileal crypt depth in comparison with the Con diet. However, no significant (*p* > 0.05) difference was observed among all the treatment groups in duodenal crypt depth. The VH:CD duodenum means in the ADS18 group were comparable to those indicated in the control and Se-Yeast groups. Se-yeast supplemented in hen diets significantly improved the VH:CD ratio in the jejunum compared to other treatments. In the ileum, the Con group had a significantly higher VH:CD ratio than the Se treatment groups.

### 3.5. Microbial Population

The effect of dietary Se supplementation on the caecal microbiota of hens is presented in Table 9. The dietary Se supplementation significantly increased (*p* < 0.05) the population of total bacteria. The organic Se supplemented hens had a significantly higher (*p* < 0.05) total bacteria population than the inorganic group, with the least in the control group hens. Higher (*p <* 0.05) populations of *Lactobacilli* spp. and *Bifidobacteria* spp. were observed in the organic (ADS18 or Se-yeast) than inorganic supplemented and control groups, respectively. Whereas means for the sodium selenite group did not significantly differ from these indicated in the Se-yeast and ADS18 groups for *Bifidobacteria* spp. population. Hens in the control group had significantly higher *Enterobacteria* spp. compared with the dietary Se supplemented groups. However, hens supplemented with organic (ADS18 or Se-yeast) Se had significantly lower (*p* < 0.05) *Escherichia coli* and *Salmonella* spp. populations than the inorganic and control hen groups, respectively. Additionally, the dietary Se supplementation did not affect the population of *Enterococcus* spp. in the caecum.

### 3.6. Volatile Fatty Acids

The effects of different dietary Se sources on the volatile fatty acids (VFA) in the cecal digesta of laying hens are presented in Table 10. Individual VFA in hen feces differed significantly (*p* < 0.05), but there was no significant difference (*p* > 0.05) in total VFA. Acetate was elevated in ADS18 and Se-yeast groups compared to the sodium selenite and control groups. Hens fed sodium selenite and controls had higher propionate, butyrate, isobutyrate, and isovalerate levels than the organic Se (ADS18 or Se-yeast) supplemented groups. No significant difference (*p* > 0.05) was observed among all the treatments for valerate; except a lower value recorded for the bacterial organic Se group. The dietary Se supplementation did not (*p* > 0.05) influence the total VFA among all the treatment groups.

## 4. Discussion

### 4.1. Laying Performance

The development and application of various Se sources in farm animals is gaining ground in the field of animal nutrition. Different forms of Se products have been extensively researched in animal nutrition, among which are Se-yeast, selenomethionine (SeMet), hydroxy-selenomethionine (OH-SeMet), and nano-Se; and currently under investigation is bacterial selenoproteins from *Stenotrophomonas maltophilia* (ADS18) bacteria. However, research on the effects of Se supplementation in the diet on laying performance or egg quality has been conflicting. The findings of this study reveal that supplementing with organic (ADS18 or Se-Yeast) or inorganic (Na_2_SeO_3_) Se influences egg production percentage, average egg weight, daily egg mass, and FCR in hens compared to those in a basal diet group.

Consistently with the recent findings on laying performance, Liu et al. [74] reported significant (*p* < 0.05) improvement in the laying rate of Roman hens supplemented with low (0.3 mg/kg selenium yeast (SY) or sodium selenite (SS)) and high (0.5 mg/kg SS) dosages of Se. Similarly, the use of nano-selenium (NS) in combination with other Se sources [75] exhibited a higher egg production percentage. Furthermore, Han et al. [76] reported that the combined supplementation of SS or SY improved egg production. However, some studies showed that laying rate was not affected by Se supplementation [77,78,79]. The positive response observed with the Se supplemented groups may have been due to the efficacy of the Se source and its bioavailability. Average egg weight was shown to be improved, especially with the Se supplemented hens. This is in agreement with the trials from Skřivan et al. [80] who used Na_2_SeO_3_ Se-enriched yeast and Se-enriched alga Chlorella in a concentration of 0.3 mg Se per kg for twenty-seven weeks. Furthermore, egg weight was significantly increased by the supplementation of Se either in SS or SY [58].

However, some authors reported no correlation between either form of Se supplementation and egg weight [18,77,81,82]. The significance for daily egg mass is in agreement with the findings of Gjorgovska et al. [83] and Zia et al. [84]. Better FCR was shown to be influenced positively by the Se supplementation in this study. Arpášová et al. [85] and Zia et al. [86] reported the same (FCR/dozen or FCR/Kg) in laying hens supplemented with selenized yeast, respectively. In the same vein, Pan et al. [87] reported an improvement due to the inclusion of Se regardless of the source. Our results showed no significant differences in average daily feed intake, live body weight change, and shell-less and broken eggs among the diets.

Based on the results of this study and the mentioned studies, the variations observed in the parameters could be attributed to differences in the metabolic pathways of inorganic and organic Se source. The speculative mechanism of the organic form of action is: that conversion of organic and inorganic selenium to readily available selenocysteine is a critical process in the production of selenoproteins, which are the major form of organic selenium that is easily absorbed by animals [88]. Selenomethionine from either selenite or selenate forms of inorganic selenium cannot be synthesized by non-ruminant animals [89,90,91,92]. The addition of these two forms of Se in the diet would require a GSH carrier as a transport medium [93].

### 4.2. Egg Quality Characteristics

Except for egg weight, eggshell thickness, egg length, width, egg shape index, surface area, albumen, and yolk weight, albumen height and egg characteristics were not affected by different Se sources. However, the observed, slight changes over the study period were numerically low and, thus, of little physiological significance. Egg quality traits can be affected by numerous factors, among which are the genetic origin of the strain, nutrition, health, environmental conditions, and facilities used. In this study, Se supplementation of different sources tended to positively affect some egg quality traits at different periods. The heavier eggs were found at certain time intervals of the study and agree with the findings of Invernizzi et al. [58] and Skřivan et al. [80]. Similarly, eggshell weight, eggshell thickness, egg shape index, and egg surface area were higher in the Se supplemented group than the basal diet group. The organic form of Se influences eggshell quality [19], although Pavlović et al. [94] disagree and reported neither the form nor the supplementation level influences eggshell quality characteristics.

The eggshell breaking strength was affected by dietary Se of any form in this study, and this agrees with the findings of Invernizzi et al. [58] and Golubkina and Papazya [95], who reported the significance of the organic form of Se in terms of its bioavailability and higher absorption by the shell glands to the shell membrane and eggshell, which in turn could be the reason for the high Se concentrations and improved shell strength. At 39 weeks of the trial, dietary Se supplementation showed a substantial difference in eggshell thickness. The findings were in agreement with those of Chantiratikul et al. [82] who used zinc-l selenomethionine and other Se sources at 0.3, 1, and 3 ppm in laying hens.

However, Baylan et al. [96] reported that Se yeast significantly improved shell weight, shell thickness, and Haugh unit positively compared to the selenite group of *Coturnix japonica*. The strength of eggshell is of prime concern to farmers in layer production units [97], as poorer eggshell strength results in an increase in the percentage of broken eggs, which in turn leads to economic consequences. Eggshell strength was maintained from the onset of lay, ended at the first phase, and declined subsequently [98]. In other findings, eggshell breaking strength was connected to higher plasma mineral content in aged hens [99]. Similarly, as hen age progresses, the eggshell strength regresses [100].

Trace mineral supplementation promotes early fusion during the early stages of shell formation, and hence improves the mechanical strength of the egg regardless of shell thickness [101]. Se yeast (1.0 mg Se/kg of dry matter, feed for 9 months) added to the diet significantly affected egg albumen weight and Haugh units in 9-month-old hens. Our results showed that the Haugh unit was not affected by either form of supplementation. It was in agreement with the findings from Liu et al. [18] and Liu et al. [74], after comparing Se sources at the low and high concentration levels.

In contrast to our results, Arpášová et al. [102] and Maysa et al. [103] reported better Haugh units in groups of birds supplemented with selenized-yeast. However, Patton [104] reported that SS or SY supplementation of 0.30 ppm did not affect Haugh unit values in eggs on day 0, 21, or 42 compared with eggs from hens fed the basal diet. Other parameters such as albumen and yolk weight and albumen height were shown to be slightly affected by the treatment diets. These results agree with the report by Skřivan et al. [80], who found hens receiving 0.3 mg/kg Se-Chlorella increased albumen weight from 38.58 to 41.27 g, yolk weight from 15.39 to 16.00 g, albumen height from 7.48 to 7.96 mm, but were unaffected in yolk height. The pH of albumen and yolk remained unaffected by different Se sources in this study.

### 4.3. Selenium Concentration in Egg Yolk and Tissue

Avian eggs are among the models used to assess the absorption and retention of microminerals, including Se of varying dosages and forms [105,106]. The amount of selenium transferred to the egg is determined by the source and level of selenium in the diet [73]. Avian eggs are one of the models used to assess the absorption and retention of microminerals, such as Se, at varying dosages and forms [105,106]. The amount of selenium transferred to the egg is determined by the source and level of selenium in the diet [73]. Lu et al. [107] found higher Se concentrations in the eggs and breast tissue of laying hens fed 0.1 to 0.4 mg/kg of Se from Se-enriched yeast than in hens fed an SS or basal diet. In addition, Liu et al. [18] found that 0.5 mg/kg of Se-yeast resulted in higher Se deposition in the egg yolk than sodium selenite in laying hens. According to Zhang et al. [108], adding Se-yeast to the diets of laying hens helps to increase Se deposition in eggs. Similarly, hens fed hydroxy selenomethionine and Se-yeast had higher yolk Se concentrations than those fed an SS or basal diet [109].

Se concentrations in breast tissue and some laying hen organs were significantly increased by dietary Se supplementation with vitamin E, Se, and their blend [110,111]; and likewise in the serum, liver, and muscle Se in growing lambs fed different levels (0.2 to 1.4 mg/kg DM) and sources (Se-met or Se-yeast and SS) of Se [112]. Han et al. [76] also reported a higher Se concentration in the serum and organs of layers fed 0.3 mg/kg Se from different Se sources. Moreover, layer chicks fed a 0.3 mg/kg diet containing both nano-Se and sodium selenite had substantially higher Se levels in tissues, organs, and serum [113]. The current study’s findings are consistent with these previous studies. The differences in Se deposition in egg yolk between Se sources may be due to their different metabolizable pathways, as inorganic Se cannot be completely metabolized to SeMet in poultry [114], and is, therefore, absorbed less, resulting in higher excretion [115]. Higher Se deposition in hens eggs fed with Se-yeast could be correlated with upregulation of the methionine (Met) metabolism gene glycine *N*-methyltransferase (GNMT) in the liver [76]. Selenoproteins (SeMet) from the liver and uterine tubes are part of egg yolk and white synthesis [4].

### 4.4. Small Intestine Histomorphology

Villus height and crypt depth are major measures of intestinal function and animal health [116]. Villi are the main components responsible for nutrient absorption in the small intestine [117], while greater villus height and continuous cell multiplication in the intestine are markers of the intestinal villi functional activity [118,119]. Increased villi height and decreased crypt depth can lead to greater absorption of nutrients, decreased gastrointestinal tract secretion, and improved growth performance [120]. Fan et al. [65] asserted that increased villus height and VH:CD ratio is positively associated with an increased turnover of epithelial cells. Despite the small intestine being known for its functions such as the digestion and absorption of nutrients, its structure and function are requisite for intestinal homeostasis, which lies upon the equilibrium between apoptosis and enterocytes proliferation [121], maintaining proper nutrient absorption and preventing bacterial translocation from the animal gut [122,123]. Previously, it has been documented that bacterial organic selenoprotein supplementation could improve intestinal morphology, although in broilers, as evidenced by increased villi height in the duodenum and ileum [124].

The present results showed that the supplementation of bacterial organic Se had a beneficial effect on villus height in all parts of the small intestine, except for the ileum. However, inorganic Se did not affect the villus height compared to a basal diet throughout all the intestinal parts. Furthermore, neither the organic nor inorganic Se supplemented fed groups had any effect on the duodenal crypt depth. These findings are consistent with Dalia et al. [124], where bacterial organic Se supplementation had a beneficial impact on villus height in all parts of the small intestine of broilers in the starter phase and the duodenum part of the finisher phase. Zamani-Moghaddam et al. [125] also found increased villus height, wider surface area, and thickness in lamina propria in all intestinal parts (duodenum and jejunum) with nano-Se supplementation in broilers, except the ileum. In addition, Ahmed et al. [126] observed a significant effect of dietary organic Se-yeast on the height of duodenum and jejunum villi in goats, and no effect on ileum villus height. Similarly, it has been reported previously that organic Se (Sel-Plex) feeding greatly increased intestinal villus height in non-challenged and reovirus-challenged broilers compared with the sodium selenite and control groups [127]. As the digestion and absorption of nutrients is the prime function of the small intestine, Zhang et al. [128] reported shorter villus height had a consequent effect on nutrient absorption by reducing the surface area, with deep crypt depth as a marker for fast tissue turnover. Fan et al. [65] postulated that shorter villus height and greater crypt depth are directly correlated with increasing enterocyte turnover.

In line with the current findings, dietary antioxidants play a significant role in gut epithelial cell protection from pro-apoptotic oxidant stress, which in turn enhances their growth and development [129,130]. The intestinal villi height and villus/crypt ratio were observed to be influenced by Se-yeast in broiler chickens, with a significant elevation of antioxidants and immune status [131]. Thus, the villus height improvement and height to crypt depth ratio in the present study with the ADS18 supplemented group might confer the significance of the organic form (exogenous antioxidant) by actively maintaining the viability of enterocytes via bioavailability of Se in intestinal glutathione peroxidase (GSH-Px2).

The efficacy of ADS18 in raising the height of the small intestine villus, especially in the duodenum portion as the primary site for Se absorption, is consistent with the work of Pesti and Combs [132]. The differences shown by sodium selenite, however, may be due to the capacity of sodium selenite to bind to the lines of epithelial tissues in the intestinal lumen, thereby being inaccessible for assimilation and transfer to tissues [133]. Furthermore, significant differences in the laying rate performance and egg quality parameters observed in hens fed organic Se form (ADS18 > Se-Yeast) could be attributed to enhanced gut integrity, via greater surface area to volume ratio in the villi.

### 4.5. Caecum Microbial Population

In general, there was an increase in the population of total bacteria, beneficial bacteria, and a decrease in pathogenic bacteria in the laying hens’ caecal digesta after supplementing with inorganic and organic Se sources. The caecum is the principal site of microbial activities in the gastro intestinal tract (GIT) and is explained as the site of an enormously complex microbial ecosystem, with specific trophic, metabolic, as well as protective, roles for the host spp [52,134,135]. It functions as a conducive environment for microorganisms to adequately utilize nutrients, as well as enhancing the animal health status and production performance with their continuous multiplication [135,136]. The productivity of the host bird is heightened by the increase in the population of the beneficial microbiota community [50]. Therefore, a practical approach to preserving host health is to increase the number of beneficial bacteria, so that they can inhibit the colonization of harmful bacteria [51,52]. Intestinal bacteria may be grouped into species with harmful or pathogenic influences on host health (*Clostridia*, *Proteus* spp., *Staphylococci* spp.); species with beneficial effects (*Lactobacilli* spp. and *Bifidobacteria* spp.); and those with dual effects (*Bacteroides* spp., *Enterococci*, and *E. coli*) [51].

Microelements as dietary components may influence the diversity of intestinal microbiota [47]. The results agree with the findings from Dalia et al. [68] who reported the potentiality of Se-enriched bacteria in modulating the caecum microbial population by boosting beneficial bacteria, as well as overwhelming the pathogenic species. Similarly, Liu et al. [137] reported an increase in *Bifidobacteria* spp. with quercetin (antioxidant) increase in laying hens. Molan et al. [138] showed that the addition of 10% and 25% Se-containing green tea (organic) resulted in a significant increase in a pure culture of *Lactobacilli rhamnosus* and *Bifidobacterium breve* population compared with China green tea containing a normal level of selenium only. Moreover, in combination with China green tea, sodium selenate growing MRS media of pure culture of *Lactobacilli rhamnosus* and *Bifidobacterium breve* enhanced their growth significantly compared to the control. Correspondingly, oral supplementation of Se-containing green tea extracts resulted in a significant increase in the population of *Bifidobacteria* spp. and *Lactobacilli* spp. in rat caecum compare to China green tea extracts [139]. A study with microalgae (*Chlorella vulgaris*) as feed supplements for laying hens resulted in an increased bacterial microbiota population in the caeca [50]. Furthermore, the bacterial culture of *Lactobacillus casei rhamnosus* supplemented with sodium selenite and cadmium (Cd) enhanced cell viability compared to cadmium culture bacteria only [140]. These support our results, as Se (organic) supplementation increased the microbe’s population of the caecum *Lactobacillus* spp. and *Bifidobacterium* spp. compared to hens supplemented with inorganic (sodium selenite) and basal diet treatments.

Furthermore, the study demonstrates the efficacy of Se supplementation; reducing the caecum population of *Escherichia coli* and *Salmonella* spp. with organic (ADS18 or Se-Yeast) Se was superior to the inorganic (sodium selenite) and control groups. In line with our findings and a trial to assess the efficacy of selenium-enriched probiotics (SeP), Lv et al. [135] reported higher *Lactobacillus* spp. fecal count and lower *Escherichia coli* in Se-enriched probiotic or probiotics compared to the sodium selenite or control group in piglets. Similarly, selenium-enriched probiotics significantly lowered the population of *E. coli* (in-vitro) and the mortality rate of mice (in vivo) inoculated with pathogenic *E. coli* [141]. However, dietary Se-yeast in chickens did not change either of the *Campylobacter jejuni* colonizations [142]. In line with the current findings, although not statistically different, Gangadoo et al. [143] reported a significant reduction of poultry pathogen (*Enterococcus cecorum*) when Se-nanoparticles were fed to mature broilers.

Trace elements (such as Se), may be effective or toxic in some groups of caecal microbiota and perhaps beneficial to some bacteria. As a result, dietary Se supplementation could provide an antioxidant function and may modulate the diversity of intestinal microbiota by suppressing the oxidative stress, which in turn, creates a better medium for the growth and multiplication of beneficial bacteria to exhibit their potential. Araúz et al. [140] and Zhang et al. [144] reported the capability of *Lactic acids* and *Bifidobacterium* spp. to incorporate inorganic (sodium selenite) Se from the growing media to their cells, with optimum growth and cell activity. In addition, lactic acid bacteria can produce antimicrobial compounds (hydrogen peroxide, lactic acid, bacteriocin-like substances), which play a vital role in the inhibition of pathogenic microbe colonization [145]. Thus, the reduction of *E. coli* and *Salmonella* spp. observed in this study could be attributed to the efficacy of organic Se (ADS18 or Se-Yeast), rather than other treatments in favor of beneficial bacteria.

### 4.6. Digesta Volatile Fatty Acids

Volatile fatty acids such as acetate, butyrate, and propionate [146] are the major metabolites in the avian gastrointestinal tract after carbohydrates fermentation by a pool of microorganisms [147], and yielded in the processing of carbohydrate (CHO) and as a marker of bacterial fermentation [148]. Dietary Se supplementation (regardless of source) to laying hens significantly increased the number of individual acids in cecal samples without affecting the total VFA. Although the relevant literature is limited, the readily available substrates for microflora digestion may be associated with an increase in some VFAs in the supplemented groups of Se. As in the present results, when studied from the impact of cecal microbial composition on odor production, Huang et al. [149] identified higher concentrations of butyrate in the cecal content of laying hens. Xun et al. [150] reported a decrease in ammonia (NH_3_-N), ruminal pH, propionate, and acetate-propionate concentration ratio, and an increase in total ruminal VFA in sheep fed 4.0 g per kg of DM nano-Se and Se-yeast. Similarly, selenocysteine is involved in the active formate dehydrogenases for the formate oxidation site [151,152], and formate (intermediate of propionate oxidation) buildup results in the accumulation of propionate due to the process of reaction inhibition mechanism [153]. Higher organic rates of anaerobic digesters are shown with Se supplementation (200 µg per L) [154]. Fecal VFAs have recently been elevated in laying hens exposed to high-temperatures. However, a decrease in total short-chain fatty acids (SCFA) in cecal digesta was observed with increased bee venom in broilers [155]. Whereas, there were no changes in the digesta VFAs of quails fed different levels of postbiotic [156]. In poultry, the production of volatile fatty acids occurs precisely in the ceca of birds that received a ration mixture [157]. Furthermore, certain inherent microbes have the potential ability to ferment undigested carbohydrates into, among other things, volatile fatty acids such as acetate, propionates, and butyrate [158]. To explain the effect of Se on volatile fatty acid production and volatile fatty acids in laying hens, further studies involving molecular approaches for examining the microbial population in response to dietary Se are required.

## 5. Conclusions

In conclusion, the results of the current study’s findings show that different dietary Se sources affected the laying hen’s production performance. The dietary organic Se (ADS18 and SY) supplemented groups increased the most in terms of laying rate, average egg weight, daily egg mass, FCR, and live body weight (LBW). However, dietary Se source had no effect on egg quality (external and internal) parameters, with the exception of eggshell breaking strength and Haugh unit, which were substantially improved with organic (ADS18 or Se-yeast) Se-fed hens compared to the control group. Hens fed dietary organic (ADS18 or Se-yeast) Se-treatments had higher Se concentrations in their egg yolks and breast tissue than the inorganic and basal diet groups. Se from bacterial selenoprotein improves intestinal integrity by increasing the height of the villus, which allows for faster nutrient assimilation and utilization compared to an inorganic (sodium selenite) source. Beneficial microbes were increased in the caecum of dietary Se supplemented hen microbiota, while harmful microbes were decreased. The individual cecal volatile fatty acids, but not total VFA, were affected by dietary Se supplementation in the laying hens. The current study did not address the safety and toxicity aspect of bacterial organic Se (ADS18), but it was noted as important for future research.

## Figures and Tables

**Table 1:** Ingredient compositions and calculated nutrient levels of the basal diet (on dry matter basis).

Ingredients	Laying
Corn (QL)	44.00
Soybean Meal (QL)	29.00
Wheat Pollard (QL)	11.00
CPO (QL)	3.50
l-Lysine	0.10
dl-Methionine	0.25
Dicalcium Phosphate (18%)	2.00
Calcium Carbonate	7.70
Choline Chloride	0.10
Salt	0.35
Mineral Mix *	0.60
Vitamin Mix **	0.60
Antioxidant ***	0.40
Toxin Binder ****	0.40
Total	100
Calculated Composition	
Metabolizable Energy Kcal/kg	2761.24
Protein (%)	17.66
Fat (%)	5.3
Fiber (%)	3.98
Calcium (%)	3.65
Total Phosphorus (%)	0.88
Av. Phosphorus for Poultry (%)	0.48

* Mineral premix supplied (per kg of diet): copper 15 mg, zinc 120 mg, iron 120 mg, manganese 150 mg, iodine 1.5 mg, and cobalt 0.4 mg. ** Vitamin premix supplied (per kg of diet): Vitamin A (retinyl acetate) 10.32 mg, vitamin E (dl-tocopherol acetate) 90 mg, cholecalciferol 0.250 mg, vitamin K 6 mg, cobalamin 0.07 mg, thiamine 7 mg, riboflavin 22 mg, niacin 120 mg, folic acid 3 mg, biotin 0.04 mg, pantothenic acid 35 mg, and pyridoxine 12 mg. *** Antioxidant contains butylated hydroxyanisole (BHA). **** Toxin binder contains natural hydrated sodium calcium aluminum silicates to reduce the exposure of feed to mycotoxins. Feed live International Software (Nonthaburi, Thailand) was used to formulate the diets.

**Table 2:** Selenium analysis of experimental diets.

Item	Treatment Diets **			
	Con	Na₂SeO₃	Se-Yeast	ADS18
Analyzed Se (mg/kg) *	0.031 ± 0.01	0.312 ± 0.02	0.320 ± 0.01	0.339 ± 0.02

** Con = control, Na_2_SeO_3_ = sodium selenite; Se-yeast = Selenium yeast; ADS18 = bacterial organic Se. * The Se content was measured using inductively coupled plasma mass spectrometry (ICP.MS).

**Table 3:** Primer sequences of cecal targeting microbes in hen digesta fed inorganic and organic Se.

Target Microorganism	Primer Sequences	Size of Amplified Fragments (bp)	Sources
Total bacteria	F: CGGCAACGAGCGCAACCC	145	[67,68]
	R: CCATTGTAGCACGTGTGTAGCC		
*Lactobacillus* spp.	F: CATCCAGTGCAAACCTAAGAG	341	[67,68]
	R: GATCCGCTTGCCTTCGCA		
*Bifidobacteria* spp.	F: GGGTGGTAATGCCGGATG	440	[66,68,69]
	R: TAAGCCATGGACTTTCACACC		
*Enterococcus* spp.	F: CCCTTATTGTTAGTTGCCATCATT	144	[68,70,71]
	R: ACTCGTTGTACTTCCCATTGT		
*Enterobacteria* spp.	F: CAT TGACGTTACCCGCAGAAGAAGC	195	[66,67,68]
	R: CTCTACGAGACTCAAGCTTGC		
*Escherichia coli*	F: GTGTGATATCTACCCGCTTCGC	82	[66,67,68]
	R: AGAACGCTTTGTGGTTAATCAGGA		
*Salmonella* spp.	F: TCGTCATTCCATTACCTACC	119	[68,70]
	R: AAACGTTGAAAAACTGAGGA		

**Table 4:** Effect of dietary supplementation of different selenium sources on the production performance of laying hens (means ± SE) *.

Parameters	Dietary Treatments ^1^				*p*-Value
	Con	Na_2_SeO_3_	Se-Yeast	ADS18	
Laying Rate (%)	87.91 ± 0.90 ^c^	90.33 ± 0.64 ^b^	91.57 ± 0.57 ^a,b^	93.75 ± 1.02 ^a^	0.0003
Average Egg Weight (g)	51.56 ± 1.12 ^b^	52.63 ± 0.65 ^a,b^	52.93 ± 0.50 ^a,b^	54.64 ± 0.47 ^a^	0.0531
Daily Egg Mass (g/Hen/Day)	46.42 ± 1.64 ^b^	48.18 ± 0.80 ^a,b^	48.53 ± 0.72 ^a,b^	51.21 ± 0.66 ^a^	0.0299
Average Daily Feed Intake (g/Hen/Day)	107.35 ± 0.65	108.57 ± 0.40	108.37 ± 0.37	108.54 ± 0.32	0.2133
Feed Conversion Ratio (g of Feed/g of Egg)	2.39 ± 0.09 ^a^	2.28 ± 0.04 ^a,b^	2.25 ± 0.04 ^a,b^	2.13 ± 0.03 ^b^	0.036
LBW	1727.78 ± 10.65 ^b^	1732.32 ± 6.65 ^b^	1753.35 ± 7.25 ^a^	1750.96 ± 8.01 ^a^	0.0945
Shell-Less and Broken Egg Rate (%)	1.56 ± 0.60	2.45 ± 0.44	1.76 ± 1.12	1.11 ± 0.39	0.5976

* Data are means of 6 replicates of 36 hens per dietary treatment. ^1^ Con: basal diet feed, Na_2_SeO_3_: basal diet feed + sodium selenite, Se-yeast: basal diet + selenium yeast, ADS18: basal diet + bacterial organic Se from *Stenotrophomonas maltophilia*. LBW, live body weight. Values in the same row with different superscripts (^a,b,c^) are significantly different (*p* < 0.05) comparing the treatment effects.

**Table 5:** Effect of dietary supplementation of different selenium sources on egg external characteristics.

Parameters	Dietary Treatments ^1^				*p*-Value
	Con	Na_2_SeO_3_	Se-Yeast	ADS18	
Egg Weight (g)	41.85 ± 0.19	41.87 ± 0.11	41.61 ± 0.13	41.86 ± 0.15	0.564
Eggshell Weight (g)	5.29 ± 0.05	5.29 ± 0.06	5.23 ± 0.12	5.30 ± 0.05	0.9282
Eggshell Thickness (mm)	0.42 ± 0.01	0.41 ± 0.02	0.41 ± 0.00	0.40 ± 0.00	0.5446
Egg Length (mm)	54.02 ± 0.14	53.72 ± 0.15	53.98 ± 0.32	53.74 ± 0.13	0.6122
Egg Width (mm)	41.85 ± 0.42	41.87 ± 0.19	41.61 ± 0.22	41.86 ± 0.29	0.564
Egg Shape Index	77.45 ± 0.42	77.95 ± 0.31	77.16 ± 0.44	77.91 ± 0.44	0.4707
Egg Surface Area (cm^2^)	70.62 ± 0.45	71.19 ± 0.17	70.99 ± 0.55	71.23 ± 0.31	0.6896
Eggshell Breaking Strength N (kg/cm^2^)	26.78 ± 0.97 ^b^	28.98 ± 0.62 ^a,b^	30.77 ± 1.23 ^a^	32.07 ± 1.74 ^a^	0.0097

Data are means of 12 eggs of 36 hens per dietary treatment. ^1^ Con: basal diet feed, Na_2_SeO_3_: basal diet feed+ sodium selenite, Se-yeast: basal diet + selenium yeast, ADS18: basal diet + bacterial organic Se from *Stenotrophomonas maltophilia*. Values in the same row with different superscripts (^a,b^) are significantly different (*p* < 0.05) comparing the treatment effects.

**Table 6:** Effect of dietary supplementation of different selenium sources on egg internal characteristics.

Parameters	Dietary Treatments ^1^				*p*-Value
	Con	Na_2_SeO_3_	Se-Yeast	ADS18	
Albumen Weight (g)	19.40 ± 0.18	19.54 ± 0.64	19.54 ± 0.22	19.64 ± 0.12	0.7022
Yolk Weight (g)	19.39 ± 0.17	18.61 ± 0.66	18.57 ± 0.21	18.65 ± 0.12	0.702
Albumen Height (mm)	8.97 ± 0.07	8.88 ± 0.07	8.85 ± 0.09	8.80 ± 0.04	0.4278
Haugh Unit	52.39 ± 2.95 ^c^	55.79 ± 2.41 ^b,c^	61.60 ± 2.54 ^b^	69.86 ± 0.76 ^a^	0.0002
Yolk pH	6.34 ± 0.14	6.63 ± 0.21	6.65 ± 0.28	6.40 ± 0.17	0.6219
Albumen pH	9.01 ± 0.29	9.01 ± 0.01	9.02 ± 0.36	8.10 ± 0.11	0.8779

Data are means of 12 eggs of 36 hens per dietary treatment. ^1^ Con: basal diet feed, Na_2_SeO_3_: basal diet feed + sodium selenite, Se-yeast: basal diet + selenium yeast, ADS18: basal diet + bacterial organic Se from *Stenotrophomonas maltophilia* (ADS18). Values in the same row with different superscripts (^a,b,c^) are significantly different (*p* < 0.05) comparing the treatment effects. Notes: Dietary treatments were supplemented with 0.3 mg/kg Se from sodium selenite (Na_2_SeO_3_), selenium yeast (Se-Yeast), and *Stenotrophomonas maltophilia* bacterial protein (ADS18).

**Table 7:** Egg yolk and Meat Se concentration.

Parameters	Dietary Treatments ^1^				*p*-Value
	Con	Na_2_SeO_3_	Se-Yeast	ADS18	
Egg Yolk µg/g	0.61 ± 0.07 ^c^	1.44 ^b^ ± 0.05 ^b^	1.91 ± 0.03 ^a^	2.04 ± 0.04 ^a^	<0.0001
Meat µg/kg	48.21 ± 0.70 ^c^	53.41 ± 0.80 ^b^	82.44 ± 1.08 ^a^	84.19 ± 1.03 ^a^	<0.0001

^a,b,c^ Means with different superscripts in the same row indicate significant difference (*p* < 0.05). ^1^ Dietary treatments: Con = control; Na_2_SeO_3_ = sodium selenite; Se-Yeast = selenium yeast; ADS18 = *Stenotrophomonas maltophilia*. SEM = standard error Means.

**Table 8:** Intestinal histomorphology of layer hens supplemented with inorganic and bacterial organic protein selenium sources.

Parameters	Dietary Treatments ^1^				*p*-Value
	Con	Na_2_SeO_3_	Se-Yeast	ADS18	
Villi Height, µm					
Duodenum	648.6 ± 25.7 ^c^	690.0 ^b^ ± 24.2 ^c^	749.6 ± 29.3 ^a,b^	784.5 ± 30.8 ^a^	0.0034
Jejunum	342.5 ± 19.2 ^c^	380.6 ± 18.8 ^b,c^	413.4 ± 19.2 ^a,b^	459.6 ± 20.1 ^a^	0.0002
Ileum	358.6 ± 17.0 ^a^	236.4 ± 12.3 ^c^	279.1 ± 12.6 ^b^	296.7 ± 10.0 ^b^	<0.0001
Crypt Depth, µm					
Duodenum	151.14 ± 3.7	104.58 ± 5.6	138.71 ± 7.2	144.39 ± 7.2	0.2673
Jejunum	102.1 ± 3.7 ^a^	85.1 ± 4.1 ^b^	85.6 ± 3.8 ^b^	74.3 ± 4.2 ^b^	<0.0001
Ileum	83.8 ± 2.4 ^a^	59.5 ± 2.1 ^b^	62.4 ± 2.1 ^b^	58.0 ± 2.3 ^b^	<0.0001
Villi Height: Crypt Depth					
Duodenum	5.53 ± 0.30 ^b^	7.20 ± 0.38 ^a^	5.8 ± 0.31 ^b^	5.730 ± 0.35 ^b^	0.0044
Jejunum	4.21 ± 0.28 ^b^	4.59 ± 0.17 ^b^	5.69 ± 0.21 ^a^	4.69 ± 0.30 ^b^	0.0003
Ileum	6.36 ± 0.30 ^a^	4.05 ± 0.20 ^b,c^	4.62 ± 0.28 ^b^	3.76 ± 0.23 ^c^	<0.0001

^a,b,c^ Means with different superscripts in the same row indicate significant difference (*p* < 0.05). ^1^ Dietary treatments: Con = control; Na_2_SeO_3_ = sodium selenite; Se-Yeast = selenium yeast; ADS18 = *Stenotrophomonas maltophilia*. SEM = standard.

**Table 9:** Caecal microbial population in layer hens fed different dietary selenium sources.

Log ^10^ Copy no/g Caecum	Dietary Treatments ^1^				*p*-Value
	Con	Na_2_SeO_3_	Se-Yeast	ADS18	
T. Bacteria	8.44 ± 0.35 ^c^	9.8 ± 0.08 ^b^	10.47 ± 0.16 ^a^	10.55 ± 0.12 ^a^	<0.0001
*Lactobacilli* spp.	6.42 ± 0.28 ^b^	6.65 ± 0.09 ^b^	7.36 ± 0.16 ^a^	7.70 ± 0.23 ^a^	0.0003
*Bifidobacteria* spp.	5.67 ± 0.69 ^c^	6.18 ± 0.27 ^b,c^	7.91 ± 0.51 ^a,b^	8.83 ± 0.84 ^a^	0.0034
*Enterobacteria* spp.	5.97 ± 0.51 ^a^	4.46 ± 0.22 ^b^	4.28 ± 0.13 ^b^	4.18 ± 0.07 ^b^	0.0004
*Enterococcus* spp.	8.24 ± 0.59	8.67 ± 0.34	8.55 ± 0.20	8.23 ± 0.41	0.8074
*Escherichia coli*	6.50 ± 0.74 ^a^	5.86 ± 0.80 ^a,b^	4.21 ± 0.29 ^b^	4.28 ± 0.18 ^b^	0.0168
*Salmonella* spp.	2.88 ± 0.25 ^a^	2.57 ± 0.30 ^a,b^	2.08 ± 0.22 ^b^	2.05 ± 0.07 ^b^	0.0447

^1^ Con = control; Na_2_SeO_3_ = sodium selenite; Se-yeast = selenium yeast; ADS18 = bacterial organic Se. ^a–c^ Values within the same column with different superscript letters differ (*p* < 0.05).

**Table 10:** Effect of different dietary selenium sources on the concentration of cecal VFA (mM) in laying hens.

Organic Acids	Dietary Treatments ^1^				*p*-Value
	Con	Na_2_SeO_3_	Se-Yeast	ADS18	
Acetate	19.00 ± 1.97 ^b^	19.03 ± 0.98 ^b^	26.74 ± 1.91 ^a^	30.79 ± 1.14 ^a^	0.0047
Propionate	1.58 ± 0.09 ^b^	2.36 ± 0.06 ^a^	1.27 ± 0.09 ^c^	1.06 ± 0.06 ^c^	<0.0001
Butyrate	1.06 ± 0.08 ^b^	1.60 ± 0.05 ^a^	0.59 ± 0.06 ^c^	0.59 ± 0.03 ^c^	<0.0001
Isobutyrate	18.68 ± 0.47 ^b^	22.32 ± 0.39 ^a^	18.90 ± 1.22 ^b^	12.48 ± 0.97 ^c^	<0.0001
Valerate	0.36 ± 0.04 ^a^	0.31 ± 0.05 ^a,b^	0.23 ± 0.01 ^b^	0.12 ± 0.04 ^c^	0.0006
Isovalerate	1.27 ± 0.15 ^a^	1.32 ± 0.05 ^a^	0.81 ± 0.02 ^b^	0.70 ± 0.04 ^b^	<0.0001
Total VFA	41.95 ± 3.17	46.92 ± 3.79	48.54 ± 2.10	45.74 ± 3.48	0.3504

^a,b,c^ Means with different superscripts in the same row differ significantly (*p* < 0.05). ^1^ Con: basal diet feed, Na_2_SeO_3_: basal diet feed + sodium selenite, Se-yeast: basal diet + selenium yeast, ADS18: basal diet + bacterial organic Se from *Stenotrophomonas maltophilia*. Total VFA, total volatile-fatty acid (acetate + propionate + butyrate + isobutyrate + valerate + isovalerate).

## Data Availability

Not applicable.

## Supplementary Materials

The following are available online at https://www.mdpi.com/article/10.3390/ani11061681/s1, Table S1: Ingredient Compositions and Calculated Nutrient Levels of the basal Diet (on Dry Matter Basis).
