# Supplementary material for: Effect of Selenium Sources on Laying Performance, Egg Quality Characteristics, Intestinal Morphology, Microbial Population and Digesta Volatile Fatty Acids in Laying Hens
Source: Animals (Basel). 2021 Jun 4;11(6):1681. doi: 10.3390/ani11061681 (PMC8228612; doi:10.3390/ani11061681)
Supplement: Supplementary file 1 [file animals-11-01681-s001.zip › animals-1165349-supplementary.pdf]

## Supplementary Materials

**Table 1.** Ingredient Compositions and Calculated Nutrient Levels of the Basal Diet (on Dry Matter Basis).

| **Ingredients** | **Pre-Lay** |
|---|---|
| Corn (QL) | 49.50 |
| Soybean Meal (QL) | 23.00 |
| Wheat Pollard (QL) | 18.50 |
| CPO (QL) | 1.50 |
| L-Lysine | 0.10 |
| DL-Methionine | 0.17 |
| Dicalcium Phosphate (18%) | 2.00 |
| Calcium Carbonate | 1.60 |
| Choline Chloride | 0.15 |
| Salt | 0.35 |
| Mineral Mix * | 1.00 |
| Vitamin Mix ** | 1.00 |
| Antioxidant *** | 0.62 |
| Toxin Binder **** | 0.62 |
| **Total** | **100** |
| **Calculated Composition** | |
| Metabolizable Energy Kcal/Kg | 2760.14 |
| Protein (%) | 16.35 |
| Fat (%) | 3.48 |
| Fiber (%) | 4.27 |
| Calcium (%) | 1.32 |
| Total Phosphorus (%) | 0.10 |
| Av. Phosphorus for Poultry (%) | 0.56 |

* Mineral premix supplied (per kg of diet): copper 15 mg, zinc 120 mg, iron 120 mg, manganese 150 mg, iodine 1.5 mg, and cobalt 0.4 mg.** Vitamin premix supplied (per kg of diet): Vitamin A (retinyl acetate) 10.32 mg, vitamin E (DL-tocopherol acetate) 90 mg, cholecalciferol 0.250 mg, vitamin K 6 mg, cobalamin 0.07 mg, thiamine 7 mg, riboflavin 22 mg, niacin 120 mg, folic acid 3 mg, biotin 0.04 mg, pantothenic acid 35 mg and pyridoxine 12 mg. *** Antioxidant contains butylated hydroxyanisole (BHA). **** Toxin binder contains natural hydrated sodium calcium aluminum silicates to reduce the exposure of feed to mycotoxins. Feed live International Software (Nonthaburi, Thailand) was used to formulate the diets.
